# Phosphorus Limitation Governs N:P Stoichiometry in Semi‐Arid Shrublands: Evidence From Organ, Plant and Community Scales

**DOI:** 10.1002/ece3.73398

**Published:** 2026-04-16

**Authors:** Yang Li, Jiangchao Guo, Yongfu Chai, Mao Wang, Pengcheng Wan, Ming Yue

**Affiliations:** ^1^ Xi'an Botanical Garden of Shaanxi Province Institute of Botany of Shaanxi Province Xi'an China; ^2^ Shaanxi Engineering Research Centre for Conservation and Utilization of Botanical Resources Xi'an China; ^3^ Key Laboratory of Resource Biology and Biotechnology in Western China (Ministry of Education) Northwest University Xi'an China; ^4^ College of Grassland and Environment Sciences Xinjiang Agricultural University Urumchi Xinjiang China

**Keywords:** ecological stoichiometry, multi‐scale analysis, phosphorus limitation, precipitation gradient, shrubland ecosystem

## Abstract

Understanding plant community adaptation in semi‐arid ecosystems is crucial for predicting their resilience under ongoing climate change. We conducted a multi‐scale investigation of nitrogen (N) and phosphorus (P) stoichiometry in 28 shrub ecosystems in northwestern China, a fragile region facing increasing aridity. Our analyses spanned plant organs, plant individuals, and the entire community, encompassing shrubs, herbs, litter, and soils. The community baseline stoichiometry averaged 12.18 g/kg N, 0.84 g/kg P, and an N:P ratio of 16.64. Phosphorus limitation was evident across all biological scales: foliar N:P ratios depended more on P than on N availability, and soil P stoichiometry correlated with multi‐scale N:P patterns. Vegetation displayed coordinated adaptive nutrient‐use strategies, with photosynthetic tissues having 49%–253% higher N or P concentrations than non‐photosynthetic organs, and dominant shrubs exhibiting 37% higher foliar P than accompanying species. Mean annual precipitation (MAP) regulated plant community N:P stoichiometry through dual pathways: a direct effect on soil N:P ratios and an indirect effect mediated by vegetation restructuring. Our findings demonstrated that precipitation was the overarching environmental factor that shaped and modulated phosphorus limitation in these ecosystems, with soil P availability acting as the proximate limiting factor. This multi‐scale perspective elucidated how plant communities adapt stoichiometrically to environmental constraints, offering a mechanistic basis for incorporating P limitation into the restoration of vulnerable dryland ecosystems.

## Graphical abstract

The multivariate effects of climate, soil nutrient status, community traits on the N:P stoichiometry of the arid shrub community. (a) N content (b) P content (c) N:P ratio.

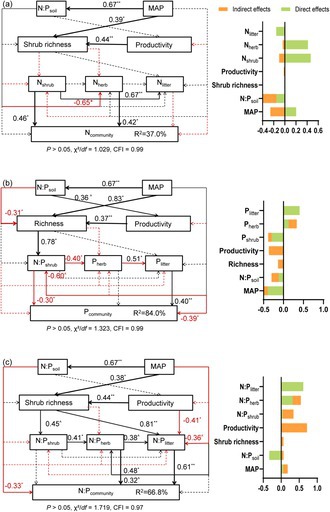

## 1 Introduction

Ecological stoichiometry provides fundamental insights into how organisms adapt to environmental constraints and regulate nutrient cycling across ecosystems (Elser et al. 2000; Sterner and Elser 2003). In terrestrial ecosystems, plant nitrogen‐to‐phosphorus (N:P) stoichiometry reflects evolutionary trade‐offs between growth, resource acquisition, and stress tolerance, particularly in resource‐limited conditions (Koerselman and Meuleman 1996; Reich et al. 2010; Han et al. 2011). Arid ecosystems present unique challenges where low precipitation restricts nutrient mobility (Helfenstein et al. 2018), while high temperatures accelerate mineralization losses (Delgado‐Baquerizo et al. 2013). Understanding such elemental balancing mechanisms is critical for predicting ecosystem responses to global change.

Beyond organismal and ecosystem‐level connections (Ren et al. 2016; Yu et al. 2020), plant N:P stoichiometry simultaneously encodes resource requirements and environmental limitations (Koerselman and Meuleman 1996; Yoshihara et al. 2010; Bradshaw et al. 2012; Hu et al. 2018; Bai et al. 2012). However, conventional studies focusing on isolated components such as leaves often ignore the interactions among ecosystem compartments (Liu and Wang 2021; Fang et al. 2025). Recent advances have deepened our understanding of plant community‐level regulation through several multiple interconnected pathways (Shen et al. 2019). For instance, niche partitioning and species interactions collectively modulate ecosystem nutrient dynamics, as demonstrated by tight stoichiometric coupling between overstory shrubs and understory herbs in mediating productivity‐nutrient feedbacks (Ning et al. 2021; Tessema and Belay 2017). Concurrently, research has elucidated the significance of intraspecific nutrient allocation, where differential partitioning among leaves, stems, and roots reflected adaptive strategies to environmental stress (Yang et al. 2018). Furthermore, the temporal dimension of stoichiometric regulation has gained increasing attention, with litter chemistry serving as a key regulator of nutrient transfer between plants and soils during decomposition (Xiao, Guenet, et al. 2015; Xiao, Janssens, et al. 2015; Zhang, Su, and Yang 2017; Zhang, Wang, et al. 2017). These advances collectively highlight the demand for multi‐scale frameworks that integrate vertical organizational levels, from organs to ecosystems, and horizontal ecosystem compartments, encompassing plants, litter, and soils (Liu and Wang 2021). Empirical evidence supports that distinct ecosystem components (e.g., leaves, litter, roots, microbes) can exhibit divergent stoichiometric responses to environmental changes, reinforcing the value of an integrated, whole‐ecosystem perspective for reliably quantifying nutrient cycling dynamics (Shen et al. 2019).

Nevertheless, critical knowledge gaps remain regarding how climatic, edaphic, and biotic factors interact to shape stoichiometric patterns across organizational scales in resource‐limited systems. Although plant nutrient stoichiometry is known to respond to complex environmental gradients (Reich and Oleksyn 2004; Han et al. 2005), arid and semi‐arid ecosystems often display unique patterns. For instance, leaf nutrient concentrations may correlate negatively with temperature while remaining decoupled from precipitation variation (He et al. 2014). These relationships were further modulated by soil biogeochemistry, with strong linkages between soil C:N:P stoichiometry and vegetation nutrient status (Han et al. 2011; Li et al. 2020). However, recent work suggested that plant community composition rather than soil nutrient availability could act as the primary driver of ecosystem stoichiometry (Hu et al. 2020). This highlighted that biotic components introduce substantial complexity through multiple mechanisms. For instance, functional group identity exerted strong effects where legumes enhanced nitrogen availability via symbiotic fixation, while grasses elevated carbon‐to‐nutrient ratios through structural carbohydrate accumulation (Guiz et al. 2016). Additionally, diversity‐stability relationships driven by complementary resource use could shape ecosystem stoichiometry (Hautier et al. 2014; Guiz et al. 2016). Diverse communities often improve nutrient‐use efficiency and promote tighter internal nutrient cycling via ecological complementarity, thereby mitigating stoichiometric imbalances (Zhao et al. 2020; Eisenhauer et al. 2024). Importantly, the direction and magnitude of such biotic effects are context‐dependent. A striking example is that plant mixtures balanced ecosystem C:N:P ratios primarily under extreme soil nutrient conditions, emphasizing strong abiotic dependency (Chen and Chen 2021). More broadly, plant community structure and environmental factors are increasingly recognized to jointly regulate ecosystem stoichiometry (Fang et al. 2025), reinforcing the necessity to explicitly consider their interactive effects. The relative contributions of direct environmental filtering versus indirect biotic mediation remain poorly quantified, especially in water‐limited ecosystems where niche partitioning may amplify interspecific stoichiometric variation. This gap is particularly pronounced for shrub‐dominated ecosystems, a dominant dryland vegetation type, where systematic multi‐scale investigations integrating organ‐level traits and community‐level biotic interactions remain scarce.

We focused this study on semi‐arid shrublands in northwestern China. Here, “shrubland” refers to vegetation dominated by shrubs with a canopy height of < 5 m and a canopy coverage of > 30% (Chai et al. 2021). These fragile ecosystems are characterized by nutrient‐poor soils and drought‐adapted shrubs, and provide keystone functions including soil stabilization, microclimate moderation, and nutrient cycling facilitation (Su and Shangguan 2021, 2022). Despite their recognized ecological importance, systematic examinations of stoichiometric properties across organizational levels, from plant organs to whole communities, remain limited.

To address this gap, we tested two central hypotheses: (1) phosphorus limitation drives N:P stoichiometry patterns across organ, individual, and community scales; and (2) mean annual precipitation regulates community N:P ratios through dual pathways: directly by modifying soil nutrient stoichiometry and indirectly by shaping vegetation structure. Within this framework, our study aimed to (1) quantify N:P stoichiometric variation across organ, individual, and community levels in 28 shrub ecosystems; (2) identify the dominant environmental controls on stoichiometric patterns at each scale. By integrating vertical organizational hierarchies with horizontal ecosystem compartments, this work advanced a mechanistic understanding of plant adaptation and nutrient regulation in semi‐arid shrublands, with implications for conservation and restoration in vulnerable dryland ecosystems.

## 2 Materials and Methods

### 2.1 Study Area

**FIGURE 1:**
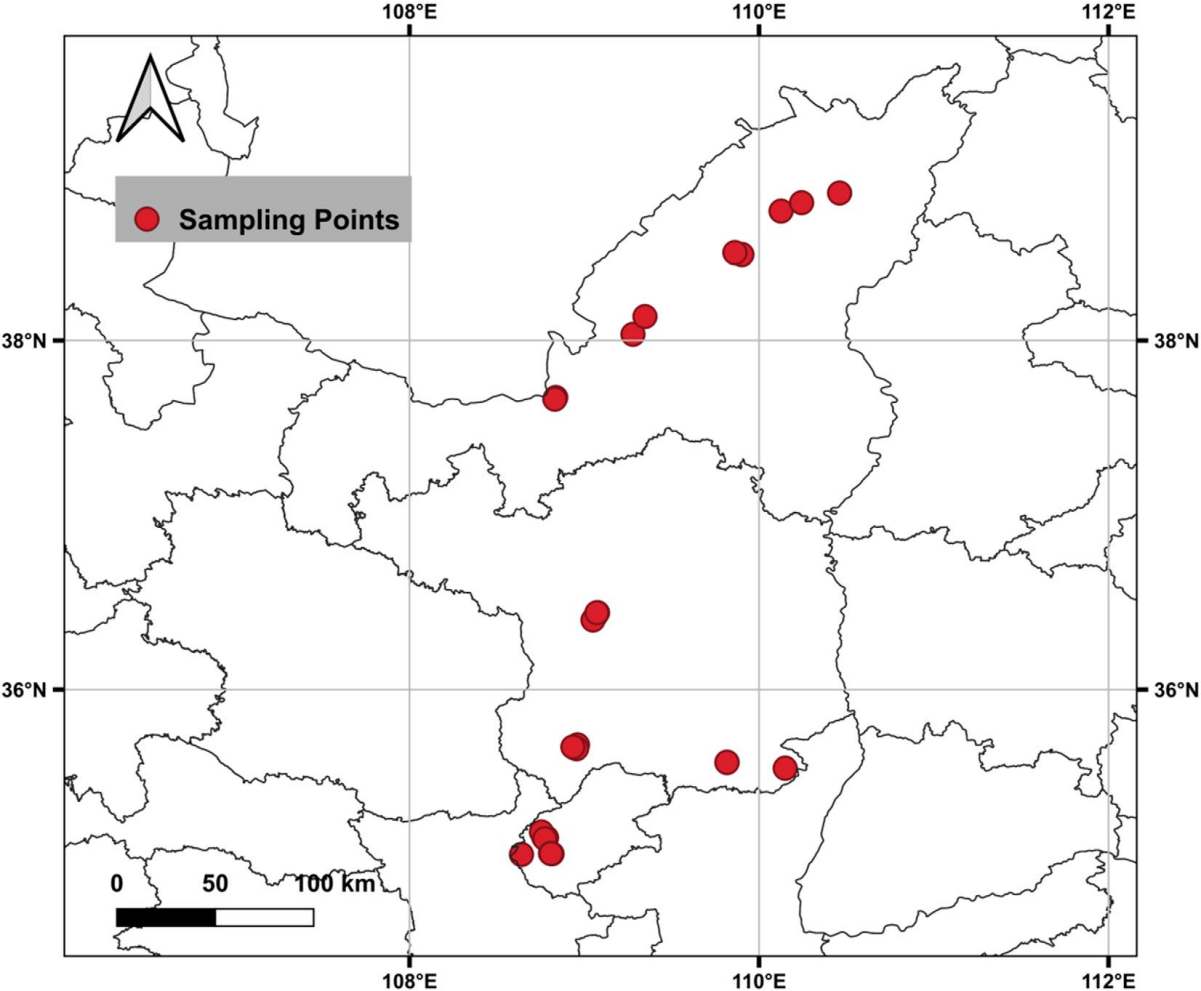
Distribution of 28 sampling sites in northwestern China.

We surveyed the main shrubland types in the semi‐arid areas of northwest China. In total, 28 shrubland communities were examined. The survey area extended from 35°06′ to 38°84′ N and from 108°6′ to 110°5′ E, with all the plots situated at elevations from 890 to 1443 m a.s.l. (Figure 1). The climatic conditions within this region exhibited significant variation, with mean annual temperatures (MAT) ranging between 3.4°C and 11.6°C, and mean annual precipitation (MAP) varying from 396 mm in the north to 634 mm in the south. The dominant plant species identified in these shrubland communities were: *Ostryopsis davidiana*, *Rosa hugonis* , *Sophora moorcroftiana*, *Caragana korshinskii* , *Hippophae rhamnoides* , and *Artemisia ordosica* (Appendix S1).

### 2.2 Data Collection

We established three random 5 m × 5 m plots at each shrubland site. Within each plot, a random 1 m × 1 m subplot was set up for detailed observation of the herbaceous layer and for litter collection.

In each 5 m × 5 m plot, every shrub individual was identified to species level. Shrub richness for the plot was calculated as the number of shrub species present. Within each 1 m × 1 m subplot, all herbaceous plants were identified. Herb richness for the subplot was calculated as the number of herbaceous species present. Total plant community richness for each study site was subsequently calculated as the total number of unique species recorded across all shrub plots and herbaceous subplots at that site.

The “Standard tree” method was employed to estimate shrub biomass. This method involves establishing species‐specific allometric relationships between easily measured plant dimensions (e.g., basal diameter, height) and total dry biomass. In the field, we selected 5–7 mature, healthy and representative adult individuals of each common shrub species (encompassing all dominant species and key accompanying plants) across a representative range of stem diameters at ground level. These selected individuals were carefully excavated to obtain their complete aboveground and belowground biomass. For belowground biomass, shrubs were excavated to the maximum depth of their root system to ensure complete recovery, which encompasses the majority of the root biomass in these semi‐arid shrublands. In the laboratory, all plant materials were oven‐dried at 65°C to constant weight and weighed. Using these data, we developed power‐law allometric equations for each common species. For rare species with insufficient individuals to build a reliable species‐specific model, we pooled data across all rare species to create a single, multispecies allometric relationship. The biomass of all unharvested shrubs in the plots was then estimated by applying the corresponding allometric equation to their measured basal diameters. Herb biomass was estimated using harvest method, where all understory plants in a subplot were excavated and divided into aboveground and belowground components. Litterfall in each subplot were collected as composite, community‐level pools from the soil surface. After drying at 65°C, the biomass was weighed and calculated. Community productivity was determined as the sum of shrub, herb and litter biomass per unit area. Total nitrogen and phosphorus in plant samples were analyzed using the Kjeldahl method (Bremner 1996) and molybdenum blue colorimetry method (Poorter and Navas 2003), respectively.

Soil samples were collected from the 0–100 cm depth of the soil profiles in each subplot, after moving the litter layer, using 5‐cm diameter stainless steel corers. Soil was collected from the following depth intervals: 0–10, 10–20, 20–30, 30–50, 50–70, and 70–100 cm (Guo et al. 2017). This stratified sampling scheme was designed to capture the vertical heterogeneity of soil properties. The finer segmentation in the top 30 cm targets the most active root zone for nutrient cycling, while the deeper intervals assess nutrient pools and potential leaching processes relevant to deep‐rooted shrubs in semi‐arid ecosystems. If the soil layer was less than 100 cm thick, we excavated down to the bedrock. Total carbon content and total nitrogen content in soil samples were determined using the elemental analysis method; the total phosphorus content in soil samples was determined using the molybdenum blue colorimetric method after perchloric acid digestion (Poorter and Navas 2003).

We acquired the data of MAT (mean annual temperature) and MAP (mean annual precipitation) at a resolution of 1 km^2^ from the “Worldclim‐Global Climate Data” (http://www.worldclim.org/).

### 2.3 Calculation of Stoichiometric Characteristics at Multiple Levels

$$\begin{array}{cc} M_{\text{shrub}}= & \sum_{i=1}^{n}\left(C_{\text{root},i}\times B_{\text{root},i}+C_{\text{stem},i}\times B_{\text{stem},i}+C_{\text{leaf},i}\times B_{\text{leaf},i}\right)/\\ & \sum_{i=1}^{n}\left(B_{\text{root},i}+B_{\text{stem},i}+B_{\text{stem},i}\right) \end{array}$$

where $M_{s\text{hrub}}$ is the shrub‐layer biomass‐weighted nutrient (N or P) concentration (g/kg),n is the number of shrub species, i is the i‐th species, $C_{\text{organ},i}$ is the directly measured nutrient concentration of a given organ, and $B_{\text{organ},i}$ is the corresponding organ biomass.

$$M_{\text{herb}}=\left(C_{\text{above}}\times B_{\text{above}}+C_{\text{below}}\times B_{\text{below}}\right)/\left(B_{\text{above}}+B_{\text{below}}\right)$$

where $M_{\text{herb}}$ is the herb‐layer biomass‐weighted nutrient (N or P) concentration (g/kg); $C_{\text{above}}$ and $C_{\text{below}}$ are the directly measured nutrient concentrations of the aboveground and belowground parts, respectively; $B_{\text{above}}$ and $B_{\text{below}}$ are the dry‐mass biomass of the aboveground and belowground parts, respectively.

$$\begin{array}{c} M_{\text{community}}=\left(M_{\text{shrub}}\times B_{\text{shrub}}+M_{\text{herb}}\times B_{h\mathrm{erb}}+C_{\text{litter}}\times B_{\text{litter}}\right)/\\ \;\left(B_{\text{shrub}}+B_{\text{herb}}+B_{\text{litter}}\right) \end{array}$$

where $M_{\text{community}}$ is the overall plant community N or P concentration, *M*_shrub_, *M*_herb_ are the biomass‐weighted nutrient concentrations calculated for the shrub and herb layers, respectively, while *C*_liiter_ is the directly measured nutrient concentration of the litter.; *B*_shrub_, *B*_herb_, *B*_liiter_ are the biomass of shrub, herb and litter.

For any given level (species, functional group, or whole community), the corresponding N:P ratio ($R_{\text{level}}$) was then calculated directly as:

$$R_{\text{level}}=\frac{M_{N,\text{level}}}{M_{P,\text{level}}}$$

where $M_{N,\text{level}}$ and $M_{P,\text{level}}$ are the biomass‐weighted N and P concentrations calculated for that specific level, as described above.

### 2.4 Data Analysis

All variables were described as mean ± standard error (SE). Data were log‐transformed as needed to meet model assumptions, and all stoichiometric ratios reported were expressed as mass ratios. One‐way ANOVA with LSD post hoc tests was used to compare N:P stoichiometry. First, we compared the major ecosystem components: shrubs, herbs, and litter. Second, we compared organs within each plant functional group: for dominant and accompanying plants, we compared leaves, stems, and roots; for herbs, we compared aboveground and belowground parts. Third, we compared the three functional groups using the same organ (e.g., leaves). Moreover, Pearson correlation analysis was used to examine the relationships between N content, P content and N:P rations in the photosynthetic organs of dominant plants, accompanying plants, and herbs, respectively. Correlations are reported as the coefficient *r* with statistical significance set at *p* < 0.05.

Fourteen factors were categorized into three groups: climate factors (MAT, MAP), soil nutrients status (soil C:N:P stoichiometry), and plant community traits (shrub richness, herb richness, richness, shrub biomass, herb biomass, and productivity). First, linear regressions with stepwise were performed to test the individual correlations between plant N:P stoichiometry and each of the fourteen factors, respectively. Subsequently, a variance partitioning analysis (VPA) was conducted based on these three predefined groups to quantify their unique and combined explanatory contributions to the variation in plant N:P stoichiometry.

Building upon the results of linear regressions and VPA, as well as established ecological principles, a Structural Equation Model (SEM) was constructed to test specific causal pathways. Following the practice of related studies, our SEM was designed to disentangle the direct and indirect effects of key variables on plant community C:N:P stoichiometry and to explicitly test our hypothesized predictions based on an integrative multivariate causal network (Table 1). The variables included in the final SEM were selected from the comprehensive set through a principled process: (a) prioritizing variables showing significant correlations and high relative importance; (b) ensuring low multicollinearity within each conceptual construct by calculating Variance Inflation Factors; and (c) iteratively refining the model to achieve parsimony and statistical identification. The final model demonstrated an acceptable/good fit to the data: *p* > 0.05, 1 < χ^2^/df < 3, and the comparative fit index (CFI) > 0.90.

**TABLE 1:** Hypothesized pathways, mechanisms, and supporting references in the SEM.

Pathway	Hypothesized mechanism
MAP→N:P_soil_	MAP regulates soil N:P by modulating soil water balance and chemical properties, thereby altering the migration capacity and biogeochemical fractions of nitrogen and phosphorus (He et al. 2014; Li et al. 2020, 2023; Zhang et al. 2020)
MAP→richness	MAP regulates plant richness by affecting regional total water availability (Mo et al. 2024)
MAP→productivity	MAP enhances soil water availability and nutrient mineralization, thereby promoting plant growth and raising productivity in arid regions (Guo et al. 2012)
MAP→plant stoichiometry	MAP affects soil nutrient availability and plant physiological regulation, thereby influencing C:N:P stoichiometry of plants (Chen et al. 2013; Li et al. 2023)
N:P_soil_ → Richness	Soil N:P imbalance drives species loss (Ma et al. 2021; Ning et al. 2021)
N:P_soil_ → productivity	Soil N:P imbalance affects productivity (Crawley et al. 2005)
N:P_soil_ → plant stoichiometry	Soil N:P ratio regulates plant nutrient uptake and community composition, thereby shaping the C:N:P stoichiometry of plant communities (Liu and Wang 2021; Ning et al. 2021)
Productivity→richness	More productive ecosystems host more diverse plant species (Gillman et al. 2015)
Richness→plant stoichiometry	Increasing plant diversity shifts stoichiometry by altering resource use strategies and interspecific nutrient partitioning (Chen and Chen 2021)
Shrub/herb/litter stoichiometry→community stoichiometry	Different ecosystem components show distinct stoichiometric responses (Shen et al. 2019)

All statistical analyses were conducted using the following software: One‐way ANOVA was performed with SAS 8.1 (SAS Institute Inc., Cary, NC, USA); correlation analysis was carried out using Origin 9.2; stepwise linear regression was implemented in IBM SPSS Statistics 20; variance partitioning analysis (VPA) was performed using the ‘vegan’ package in R; and the SEM analysis was conducted using IBM SPSS Amos 22. Figures were prepared using GraphPad Prism 8 and Microsoft PowerPoint 2017.

All analyses were conducted with SAS software 9.3 (SAS Institute Inc., Cary, NC, USA), Origin 9.2, and R program. Figures were generated by GraphPad Prism and PowerPoint 2017.

## 3 Results

### 3.1 The C:N:P Stoichiometry in the Semi‐Arid Shrub Community

**FIGURE 2:**
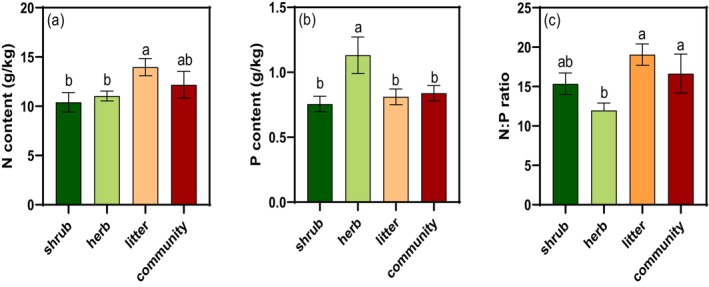
The N:P stoichiometry in different components in the arid shrub community. (a) N content (b) P content (c) N:P ratio. The same letter indicated that there was no significant difference between two components.

The semi‐arid shrub community showed mean N and P contents of 12.18 ± 1.35 and 0.84 ± 0.06 g/kg, respectively, with an overall N:P ratio of 16.64 ± 2.47 (Figure 2). Significant stoichiometric variation was detected among functional components (all *p* < 0.05). Nitrogen was highest in litter, whereas phosphorus peaked in herbs. Accordingly, the N:P ratio decreased progressively from litter (19.05) to shrubs (15.35) to herbs (11.98; Figure 2c).

**FIGURE 3:**
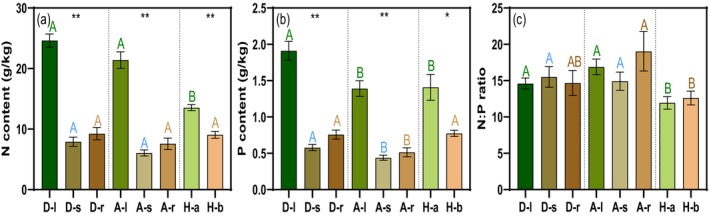
The N:P stoichiometry in different organs of dominant plants, accompanying plants, and herbs. (a) N content (b) P content (c) N:P ratio. D‐l, D‐s, and D‐r were represented leaves, stems, and roots of dominant plants, respectively; A‐l, A‐s, and A‐r were represented leaves, stems, and roots of accompanying plants, respectively; H‐a and H‐b were represented aboveground components and belowground components of herbs, respectively. The same letter indicated that there was no significant difference in the same organs among dominant plants, accompanying plants, and herbs. Asterisk indicated that there was significant difference among different organs in dominant plants, accompanying plants, or herbs, respectively. *< 0.05, **< 0.001.

**FIGURE 4:**
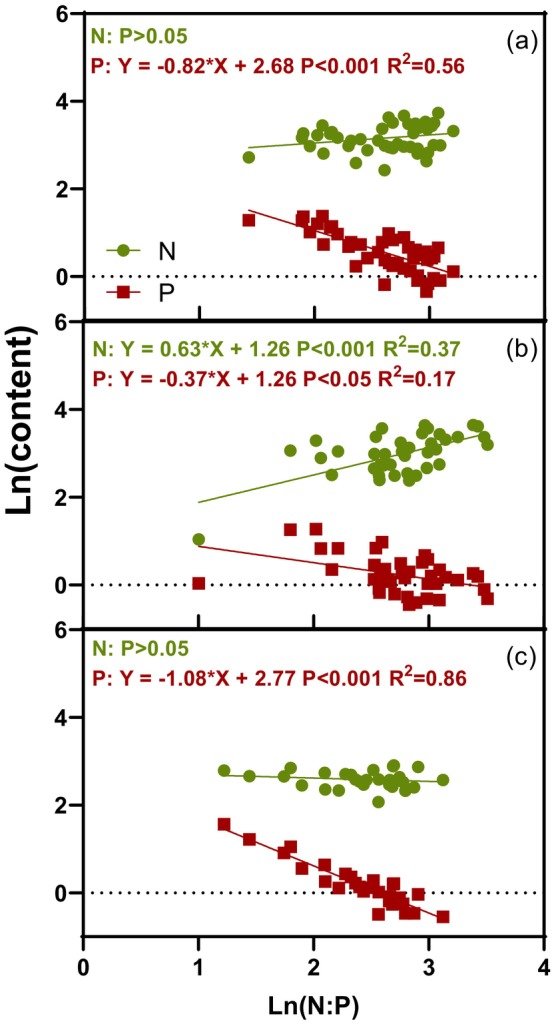
The correlations between ln(N:P) and ln (N) or ln (P) in photosynthetic organ of (a) dominant plants, (b) accompanying plants, and (c) herbs.

Plant functional types exhibited distinct organ‐level nutrient allocation patterns. Across shrub species, dominant individuals consistently showed higher phosphorus concentrations in roots, stems, and leaves than accompanying individuals (all *p* < 0.05; Figure 3b), whereas no such difference was detected for nitrogen (all *p* > 0.05; Figure 3a). A marginally higher foliar N:P ratio was observed in accompanying shrubs (*p* = 0.08; Figure 3c). Within each functional group, leaves accumulated the highest N and P concentrations, while herbs allocated more nutrients to aboveground than belowground tissues (all *p* < 0.001; Figure 3a,b). N:P ratios were largely conserved across organs within groups (all *p* > 0.05; Figure 3c), and variation in foliar N:P of dominant shrubs and aboveground herb N:P was primarily driven by phosphorus rather than nitrogen availability (both *p* < 0.001 for P effects; Figure 4a,c).

### 3.2 Influencing Factors and Their Relative Contributions to N:P Stoichiometry

**FIGURE 5:**
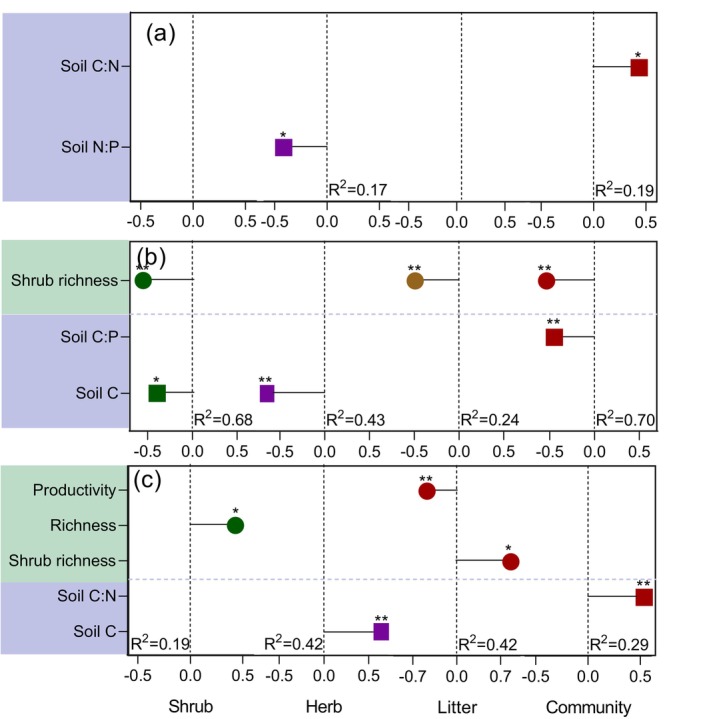
The standardization coefficient for the effects of climate, soil nutrient status, community traits on the N:P stoichiometry of community components in the arid shrub community was determined using stepwise linear regressions. (a) N content (b) P content (c) N:P ratio. All regression equations follow the form $Y=\beta_{1}X_{1}+\beta_{2}X_{2}+\ldots +\beta_{n}X_{n}$ (Y: Respective stoichiometric index; $X_{n}$: Significant explanatory factors; $\beta_{n}$: Standardized coefficients), with adjusted *R* ^2^ values represented the coefficient of determination. *< 0.05, **< 0.001.

Stepwise linear regression identified distinct environmental and community predictors for different stoichiometric components. Community‐level N was associated primarily with soil C:N ratio, whereas community‐level P was associated with both soil C:P ratio and shrub richness (both *p* < 0.05; Figure 5a,b). The community N:P ratio was mainly explained by soil C:N ratio (*p* < 0.05; Figure 5c). For individual functional components, soil N:P ratio was linked to herb N content, while soil C content and shrub richness individually and collectively explained P variation in shrubs, herbs, and litter (Figure 5a,b). The N:P ratios of shrubs, herbs, and litter were each associated with different predictors—namely, richness, shrub richness, productivity, and soil C content, respectively (all *p* < 0.05; Figure 5c).

**FIGURE 6:**
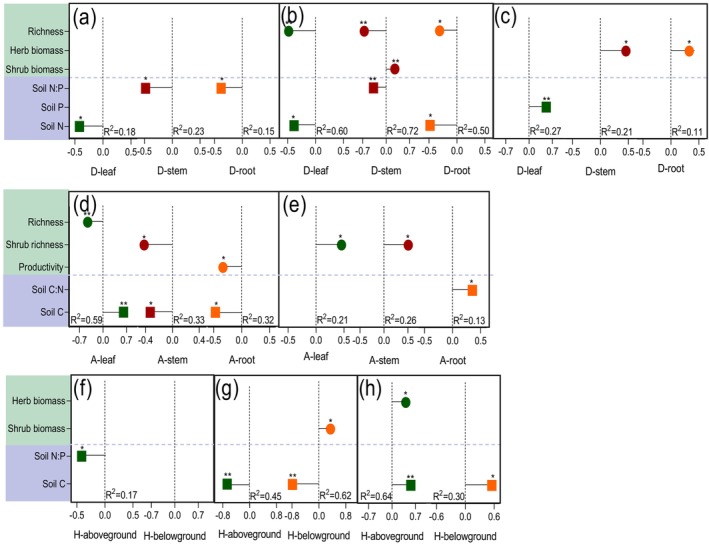
The standardization coefficient for the effects of climate, soil nutrient status, community traits on the N:P stoichiometry of different organs of dominant plants, accompanying plants, and herbs in the arid shrub community was determined using stepwise linear regressions. (a) N content of dominant shrubs (b) P content of dominant shrubs (c) N:P ratio of dominant shrubs (d) P content of dominant shrubs (e) N:P ratio of dominant shrubs (f) N content of herbs (g) P content of dominant herbs (h) N:P ratio of dominant herbs. *R* ^2^ values represented the coefficient of determination. All regression equations follow the form $Y=\beta_{1}X_{1}+\beta_{2}X_{2}+\ldots +\beta_{n}X_{n}$ (Y: Respective stoichiometric index; $X_{n}$: Significant explanatory factors; $\beta_{n}$: Standardized coefficients), with adjusted *R* ^2^ values represented the coefficient of determination. * < 0.05, ** < 0.001.

Stepwise linear regression revealed that organ‐level N:P stoichiometry was governed by distinct predictors across functional groups and organs (Figure 6). Nitrogen concentrations in dominant and herbaceous plants were primarily linked to soil N or soil N:P ratio (all *p* < 0.05, Figure 6a,f). Phosphorus allocation patterns differed by functional type. In dominant shrubs, leaf, stem, and root P were each associated with combinations of richness, shrub biomass, soil N, and soil N:P ratio (all *p* < 0.05, *R* ^2^ = 0.50–0.72; Figure 6b). In accompanying shrubs, leaf and stem P were explained by soil C and richness or shrub richness (both *p* < 0.05, *R* ^2^ = 0.33–0.59; Figure 6d), while root P was linked to soil C and productivity (*p* < 0.05, *R* ^2^ = 0.32; Figure 6d). For herbs, both aboveground and belowground P were associated with shrub biomass and/or soil C (all *p* < 0.05, *R* ^2^ = 0.45–0.62; Figure 6g). The N:P ratios of different organs also showed functional‐group‐specific controls: they were linked to soil P or herb biomass in dominant shrubs, to soil C:N ratio and shrub richness in accompanying shrubs, and to soil C and/or herb biomass in herbs (all *p* < 0.05, *R* ^2^ = 0.30–0.64; Figure 6c,e,h).

**FIGURE 7:**
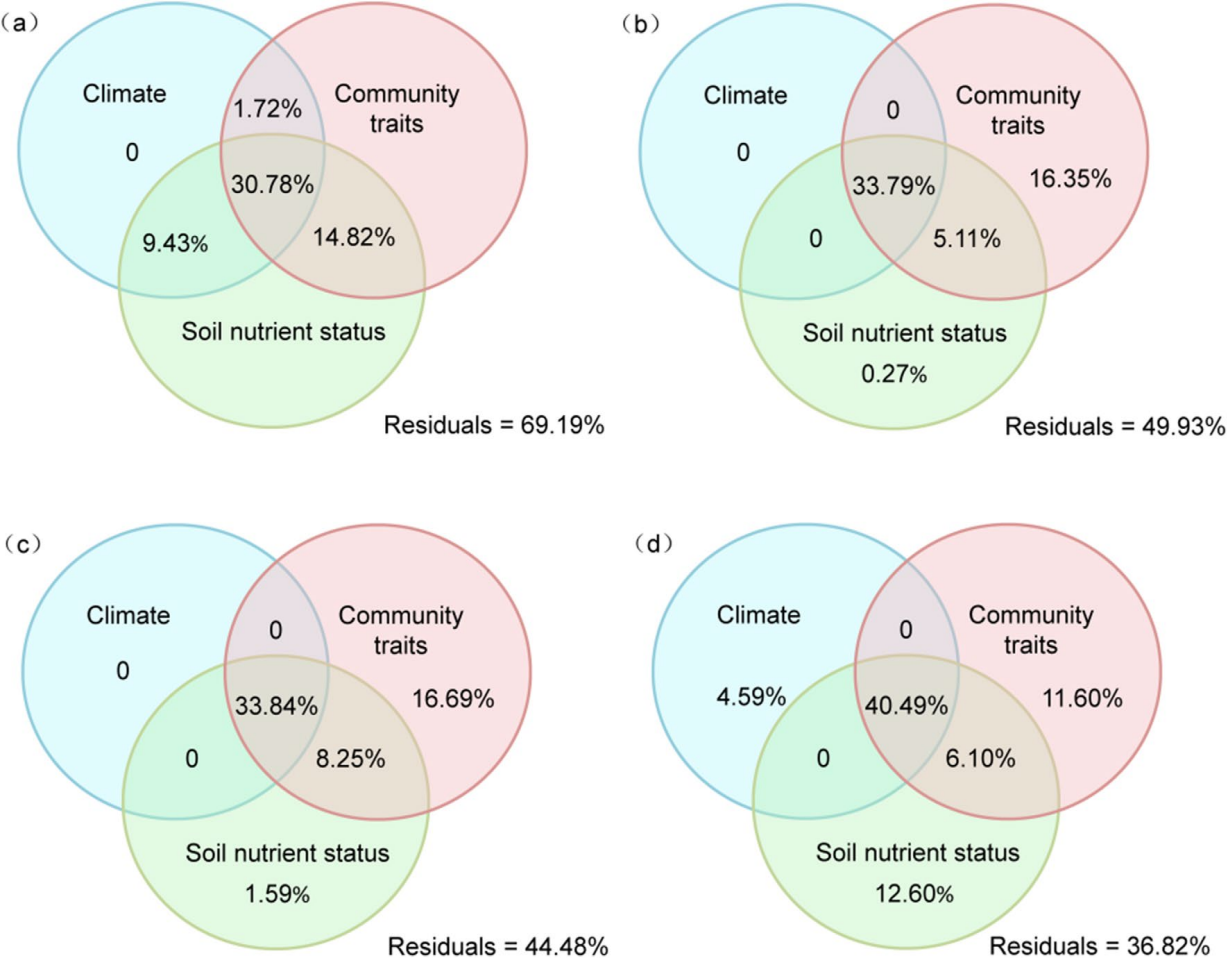
Variation partitioning in C:N:P stoichiometry of the arid shrub community.

To assess stoichiometric drivers across biological scales, variance partitioning was conducted at four levels: leaf, dominant plant, shrub layer, and whole community (Figure 7). At the leaf level, no factor group contributed independently; variation was predominantly explained by two‐way (25.97%) and three‐way (30.78%) interactions (Figure 7a). At the plant and shrub levels, plant community traits showed strong independent contributions (16.35% and 16.69%), while soil nutrients had minimal unique effects (0.27% and 1.59%). The interaction between community traits and soil nutrients explained an additional 5.11% (plant) and 8.25% (shrub), and the shared three‐way effect accounted for about 34% at both levels (Figure 7b,c). At the community level, independent effects of climate (4.59%), plant traits (11.60%), and soil nutrients (12.60%) were all detectable, yet the shared three‐way interaction was the largest component (40.49%), followed by the trait–soil interaction (6.10%, Figure 7d).

### 3.3 The Pathway of Different Factors on the N:P Stoichiometry

**FIGURE 8:**
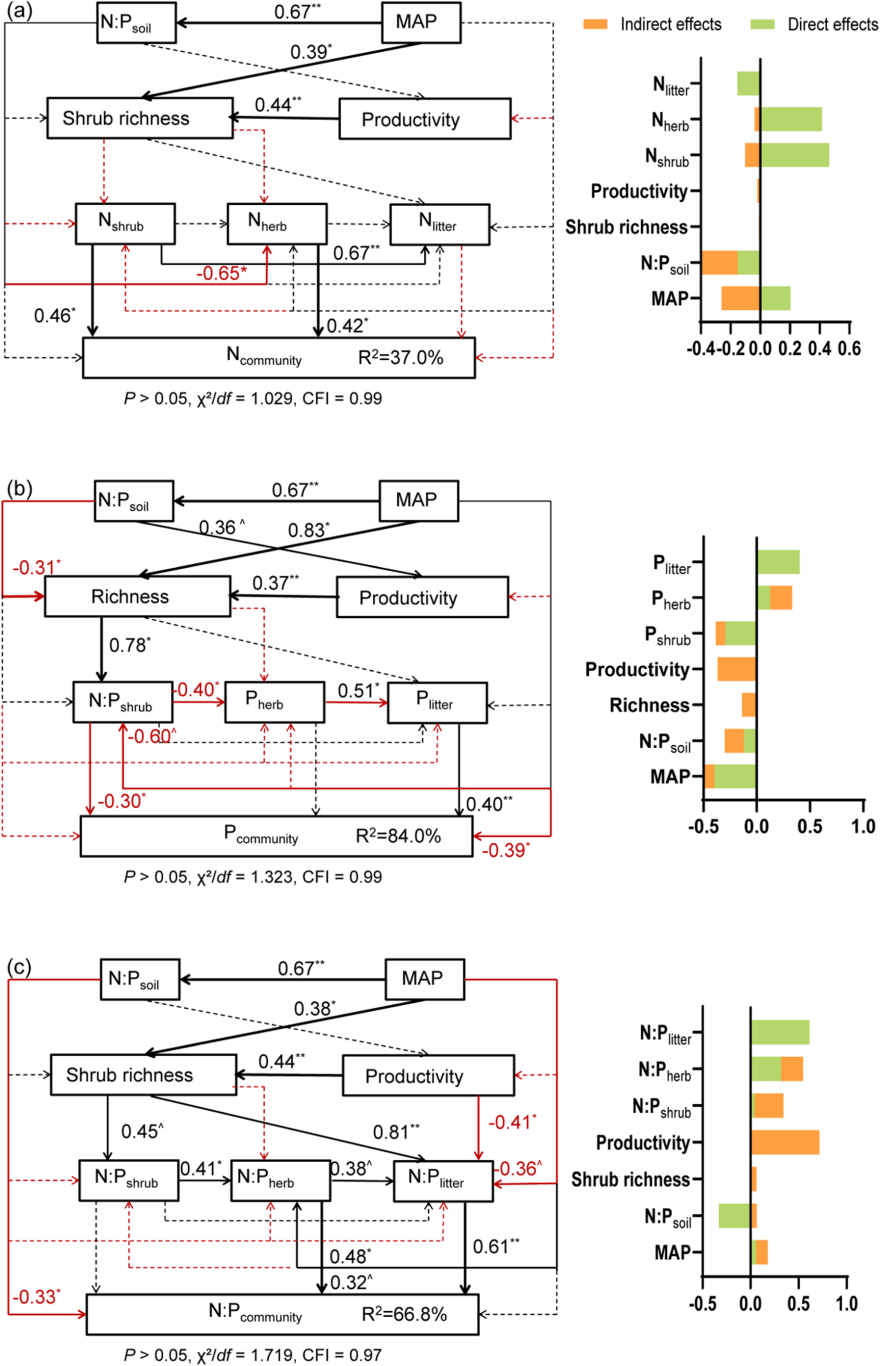
Pathway model of the multivariate effects of climate, soil nutrient status, plant community traits on the N:P stoichiometry of the arid shrub community. (a) N content (b) P content (c) N:P ratio. Solid red arrows indicated significantly negative relationships. Solid black arrows represented significantly positive relationships. Dashed lines (both red and black) indicated that paths were insignificant. Numbers connected arrows were standardized path coefficients (^ˆ^ *p* < 0.10; *p < 0.05; **p < 0.001). *R* ^2^ values represented the percentages of the variance explained by the model. *n* = 28.

The final structural equation models (SEMs) demonstrated excellent fit to the data (*p* > 0.05, 1 < χ^2^/df < 3, CFI > 0.90) and revealed hierarchical pathways influencing ecosystem stoichiometry (Figure 8, *n* = 28). Mean annual precipitation (MAP) emerged as a key driver. It directly influenced soil N:P (*p* < 0.001, *r* = 0.67), which in turn affected herb N and ultimately community N (both *p* < 0.05), explaining 37.0% of its variation (Figure 8a). MAP also shaped species richness (*p* < 0.05, *r* = 0.83) and shrub N:P (*p* = 0.05, *r* = −0.60), both directly and indirectly via richness, and together with shrub N:P and litter P explained 84.0% of the variance in community P (Figure 8b). Additional MAP‐driven pathways operated through shrub richness, herb N:P, and litter N:P. Shrub richness further modulated community productivity and litter N:P. Community N:P was directly controlled by soil (*p* < 0.05, *r* = −0.33), herb (*p* < 0.10, *r* = 0.32), and litter (*p* < 0.001, *r* = 0.61) N:P, which jointly accounted for 66.8% of its variance (Figure 8c).

## 4 Discussion

Our multi‐scale investigation of semi‐arid shrub ecosystems demonstrated consistent variation in N:P ratios across hierarchical biological scales, ranging from preferential nutrient allocation to photosynthetic organs at the tissue scale to competitive dominance and species‐specific nutrient strategies at the community level. Our results identified phosphorus availability as the primary limiting factor across these ecosystems. Importantly, we detected a dual regulatory pathway by which climate shapes plant community stoichiometry, operating through both direct modification of soil N:P stoichiometry and indirect vegetation‐mediated pathways. These findings collectively advance our understanding of how abiotic constraints and biotic interactions jointly govern ecosystem nutrient stoichiometry in water‐limited environments.

### 4.1 Multi‐Scale Stoichiometric Patterns and Ecological Adaptation

The observed N:P stoichiometric variations across biological scales revealed adaptive resource optimization strategies employed by semi‐arid shrubs under severe environmental constraints. Photosynthetic tissues exhibited metabolic prioritization through enhanced nitrogen and phosphorus allocation, a widespread adaptive response to nutrient limitation in dryland systems (Qin et al. 2016; Tian et al. 2017; Zhang et al. 2019; Luo et al. 2021). Such differential nutrient partitioning among organs reflected functional adaptation to harsh habitats, where elevated nutrient concentrations in foliage support efficient carbon assimilation and stress tolerance (Drenovsky et al. 2010). As structural and resource‐acquiring tissues, stems and roots generally maintained lower nutrient concentrations, partly due to dilution effects associated with greater biomass investment in these tissues (Kerkhoff et al. 2006; Zhang, Su, and Yang 2017; Zhang, Wang, et al. 2017). This pattern was further supported by lower C:N and C:P ratios in leaves relative to other organs (Shi et al. 2017). Such organ‐level stoichiometric patterns aligned with theoretical expectations of scale‐dependent stoichiometric regulation in nutrient‐limited systems (Niklas and Cobb 2005; Li et al. 2017; Luo et al. 2021). Our observations corroborated previous findings that desert shrubs preferentially enrich nutrients in photosynthetic organs rather than structural or absorptive tissues (He et al. 2016; Luo et al. 2021).

Examining N:P stoichiometry across community components provided further insight into adaptive strategies among coexisting species under nutrient limitation (Zhang et al. 2011). In this study, dominant shrubs displayed higher foliar phosphorus concentrations than accompanying species, suggesting more efficient phosphorus acquisition or utilization in this phosphorus‐limited environment. Conversely, accompanying plants exhibited both higher foliar N:P ratios and lower absolute phosphorus concentrations. This stoichiometric signature, characterized by high N:P coupled with low phosphorus concentrations, aligned with traits of stress‐tolerant (S) species within the C‐S‐R strategy framework (Grime 2001; Güsewell 2004). In such species, elevated N:P ratio reflected slow growth rate and efficient internal phosphorus cycling (Güsewell 2004), both critical adaptations to resource‐limited environments. This stoichiometric divergence likely reinforced competitive hierarchy, with dominant shrubs sustaining greater growth through superior phosphorus‐use efficiency. Comparisons between overstory shrubs and understory herbs revealed further dimensions of nutrient adaptation. Although herbaceous plants typically exhibit higher tissue nutrient concentrations (Niklas and Cobb 2005), shrubs in our system maintained foliar N and P levels comparable to or exceeding those of herbs. This pattern suggested that shrubs prioritize nutrient allocation to photosynthetic tissues, thereby enhancing photosynthetic efficiency and growth potential under arid stress (Yu et al. 2010; Bai et al. 2019). Such conservative nutrient‐use strategies represented key adaptations to environments where resource availability strongly constrains productivity.

Litter stoichiometry provided insights into nutrient conservation and cycling potential at the ecosystem scale. In this study, we collected composite surface litter samples within each plot, representing the standing pool of senesced material available for decomposition and nutrient return. Although this approach integrated litter at varying stages of senescence and early decomposition, application of widely used comparative thresholds (Killingbeck 1996; Yang et al. 2018) enabled a robust ecosystem‐level assessment of relative nitrogen and phosphorus conservation. Our results showed that community‐wide litter nitrogen concentrations remained above 1.00%, while phosphorus concentrations fell below 0.08%. Base on the threshold framework, this pattern indicated more complete phosphorus than nitrogen conservation prior to and during litter formation. According to the relative resorption hypothesis, this implied that the community may be subject to phosphorus limitation (Han et al. 2013). Correspondingly, elevated litter N:P reflected strong ecosystem‐scale retention of the limiting nutrient (phosphorus) in these semi‐arid shrublands. These stoichiometric patterns had direct implications for nutrient cycling, as they govern the quality and rate of nutrient return to the soil (Xiao, Guenet, et al. 2015; Zhang, Su, and Yang 2017; Zhang, Wang, et al. 2017). By linking litter stoichiometry to differential nutrient conservation, our findings advanced the general understanding of nutrient retention strategies in resource‐limited ecosystems.

Collectively, the observed variations in N:P stoichiometry across plant organs and community components provided evidence for multiple, coordinated adaptive strategies in this semi‐arid shrub ecosystem. The documented patterns included preferential nutrient allocation among organs, stoichiometric divergence between dominant and accompanying species, and distinctive litter stoichiometric signatures, all of which support mechanisms involving adaptive nutrient partitioning, competitive hierarchy formation, and differential nutrient resorption. These integrated strategies likely enhanced overall community fitness under the resource‐limited conditions that characterize semi‐arid environments.

### 4.2 Soil Phosphorus Availability as the Primary Limiting Factor

Our findings demonstrated that soil phosphorus availability served as the primary limiting factor governing ecosystem stoichiometry in these semi‐arid shrublands. This observation aligned with previous studies indicating that desert plant ecosystems in northern China were relatively phosphorus‐limited (Han et al. 2005; Luo et al. 2021). Three lines of evidence from our study supported this conclusion. Firstly, the N:P ratios in leaves and aboveground tissues, widely recognized indicators of nutrient limitation, exhibited stronger dependence on plant phosphorus content than nitrogen content across all functional groups (Figure 3). This relationship followed established stoichiometric principles, where foliar N:P ratios correlated inversely with plant phosphorus concentrations, reflecting the fundamental role of phosphorus in growth and productivity (Güsewell 2004; He et al. 2008; Mei et al. 2019). It also supported empirical evidence that variation in ecosystem N:P stoichiometry was predominantly driven by phosphorus dynamics (Shen et al. 2019). Secondly, at the community level, both stepwise regression and structural equation modeling revealed significant negative relationships between species richness and plant phosphorus content, implying intensified competition for scarce soil phosphorus under higher plant diversity. Thirdly, positive correlations between herbaceous biomass and community N:P ratios indicated intensifying phosphorus limitation with rising productivity, as plant phosphorus concentrations declined despite greater biomass production.

This phosphorus limitation stemmed from the interplay of geochemical scarcity, climatic constraints on nutrient cycling, and plant adaptation to harsh habitats. The inherently low soil phosphorus content (0.49 g/kg Figure S1), lower than the national averages of 0.65 g/kg and global averages of 0.72 g/kg (Cleveland and Liptzin 2007; Tian et al. 2010), reflected limited phosphorus supply driven by slow mineral weathering and deposition. This geochemical constraint was further aggravated by the arid climate of the study region, characterized by low precipitation and high evaporation (Luo et al. 2021). Plants primarily take up phosphorus in its dissolved inorganic form from the soil solution (Helfenstein et al. 2018). However, extremely low soil water availability in our study region not only restricted phosphorus diffusion and replenishment of soluble phosphorus pools (Zhang et al. 2020), but also impaired microbial activity responsible for phosphorus mineralization and biogeochemical cycling (Richardson and Simpson 2011; Pistocchi et al. 2018; Mei et al. 2019; Rafi et al. 2019). Drought conditions therefore reduced phosphorus bioavailability, slowed nutrient turnover, and weaken nutrient feedbacks between plants and soil (Huang et al. 2018). These climatic and geochemical constraints may be further amplified by increasing nitrogen deposition (Elser et al. 2007; Peñuelas et al. 2013; Li et al. 2016). Elevated nitrogen inputs stimulated plant growth and intensify plant phosphorus demand, while soil phosphorus availability remained constrained by weak weathering, low mineralization rates, and limited diffusion (Filippelli 2008; Augusto et al. 2017). This created a positive feedback loop in which increased productivity further depleted available phosphorus (Sun et al. 2017). Notably, such shifts in ecosystem N:P stoichiometry were often driven primarily by phosphorus dynamics rather than changes in nitrogen (Shen et al. 2019). Collectively, these mechanisms indicated that low soil phosphorus availability, combined with drought‐induced constraints on nutrient cycling and impaired plant phosphorus acquisition, jointly drove the strong phosphorus limitation observed in this semi‐arid shrubland ecosystem.

### 4.3 Climate as the Ultimate Driver: Direct and Indirect Pathways

Climatic factors emerged as ultimate regulators of plant community stoichiometry, primarily by modulating soil nutrient availability and community composition. Our finding that mean annual precipitation (MAP) strongly predicted soil N:P ratios aligned with global‐scale evidence that hydrothermal conditions govern soil stoichiometry through integrated effects on mineral weathering, plant nutrient demand, and microbial decomposition (Chadwick et al. 1999; Tian et al. 2010). Pearson correlation analysis further confirmed significant associations between soil C:N:P ratios and both MAT and MAP (all *p* < 0.05; Figure S1). Hydrothermal conditions influenced soil nutrient stoichiometry mainly through altering mineral weathering, plant growth and nutrient demand, and microbial decomposition rates (Tian et al. 2010; Sanaullah et al. 2012). These climate‐driven processes can lead to divergent outcomes, ranging from nutrient depletion via enhanced plant uptake to accumulation through increased organic inputs, depending on the specific ecosystem context (Arenberg and Arai 2019; Hernández‐Romero et al. 2024).

In addition to these direct geochemical pathways, climate exerted indirect control through vegetation feedbacks. Along the climatic gradients examined, hydrothermal availability enhanced community richness (Figure S2), which is consistent with broadscale biogeographic patterns (Mo et al. 2024). This enhanced diversity likely intensified belowground competition for limiting soil nutrients, particularly under low‐fertility conditions where niche partitioning can amplify small stoichiometric differences among coexisting species (Casper 1997). Such vegetation‐mediated regulation was especially pronounced in resource‐limited environments, where plant community structure itself became a key modulator of nutrient dynamics.

Collectively, these findings established climate's dual regulatory role. It acted as a direct geochemical agent shaping soil nutrient baselines and as an indirect biotic mediator influencing nutrient acquisition and competition through community restructuring. This integrated perspective provided a new framework for predicting ecosystem stoichiometric responses to ongoing aridification.

## 5 Conclusion

These findings collectively advanced a framework where climate set the template for ecosystem stoichiometry, phosphorus availability filtered community composition, and biotic interactions amplified initial stoichiometric differences. The hierarchical nature of these controls had important implications for predicting ecosystem responses to ongoing aridification. Restoration efforts should prioritize species with high P‐use efficiency while monitoring litter stoichiometry as an indicator of nutrient cycling function. Targeted phosphorus amendments may prove necessary in areas experiencing combined N enrichment and drought intensification, though such interventions require careful evaluation of long‐term ecosystem effects.

## Author Contributions

**Yang Li:** data curation (lead), methodology (equal), writing – original draft (lead), writing – review and editing (equal). **Jiangchao Guo:** data curation (equal), investigation (equal). **Yongfu Chai:** data curation (equal), investigation (equal). **Mao Wang:** investigation (equal). **Pengcheng Wan:** investigation (equal). **Ming Yue:** conceptualization (equal), methodology (equal), writing – review and editing (equal).

## Funding

This work was supported by the Applied Technology Research and Development Project of Shaanxi Academy of Sciences, 2026K‐03. the Natural Science Basic Research Plan of Shaanxi Province, 2025JC‐YBMS‐206. the Restoration Project of Mountains, Rivers, Forests, Fields, Lakes, Grasslands and Deserts in the Northern Foothills of Qinling in Shaanxi Province, 2203‐610100‐04‐05‐321562.

## Conflicts of Interest

The authors declare no conflicts of interest.

## Supporting information


**Appendix S1:** Detailed information of the 28 surveyed plant communities.

**Figure S1:** Soil C:N:P stoichiometric characteristics and their correlations with temperature and precipitation.
**Figure S2:**. The correlations among climate factors, community traits and soil nutrient stoichiometry.

## Data Availability

The data that support the findings of this study are openly available in the Dryad Digital Repository at https://doi.org/10.5061/dryad.mcvdnckdw.
