# Supplementary material for: Phosphorus Limitation Governs N:P Stoichiometry in Semi‐Arid Shrublands: Evidence From Organ, Plant and Community Scales
Source: Ecol Evol. 2026 Apr 16;16(4):e73398. doi: 10.1002/ece3.73398 (PMC13087109; doi:10.1002/ece3.73398)
Supplement: Supplementary file 1 — Appendix S1: Detailed information of the 28 surveyed plant communities. [file ECE3-16-e73398-s001.docx]

**Appendix:** Detailed Information of the 28 Surveyed Plant Communities

|  | **Longitude** | **Latitude** | **MAT**  **(℃)** | **MAP**  **(mm)** | **Dominant plant species** | **RB**  **(%)** |
| --- | --- | --- | --- | --- | --- | --- |
| 1 | E108°47′01″ | N35°09′08″ | 10.2 | 611 | *Ostryopsis davidiana* | 66.62 |
| 2 | E108°46′03″ | N35°09′21″ | 10.7 | 596 | *Vitex negundo* | 52.96 |
| 3 | E108°45′17″ | N35°11′07″ | 9.5 | 610 | *Rosa hugonis* | 74.01 |
| 4 | E108°46′27″ | N35°08′47″ | 10.5 | 609 | *Cotinus coggygria* | 14.11 |
| 5 | E108°57′40″ | N35°40′59″ | 10.2 | 558 | *Sophora davidii* | 37.15 |
| 6 | E108°57′22″ | N35°39′43″ | 9.8 | 566 | *Lonicera japonica* | 11.78 |
| 7 | E108°56′05″ | N35°40′15″ | 10.0 | 561 | *Sophora davidii* | 34.62 |
| 8 | E109°04′06″ | N36°25′41″ | 9.2 | 511 | *Ostryopsis davidiana* | 42.74 |
| 9 | E109°04′33″ | N36°26′27″ | 8.9 | 501 | *Vitex negundo* | 85.95 |
| 10 | E109°02′60″ | N36°23′53″ | 8.3 | 508 | *Hippophae rhamnoides* | 81.36 |
| 11 | E109°04′20″ | N36°26′25″ | 9.6 | 511 | *Ziziphus jujuba* | 85.38 |
| 12 | E109°49′05″ | N35°35′25″ | 10.3 | 568 | *Sophora davidii* | 52.24 |
| 13 | E109°49′23″ | N35°35′17″ | 10.2 | 568 | *Ostryopsis davidiana* | 46.76 |
| 14 | E109°49′39″ | N35°35′68″ | 9.0 | 582 | *Syringa reticulata* | 60.48 |
| 15 | E110°09′57″ | N35°33′55″ | 11.6 | 544 | *Sophora davidii* | 62.36 |
| 16 | E108°38′26" | N35°03′22" | 9.7 | 634 | *Rosa hugonis* | 44.63 |
| 17 | E108°48′55" | N35°03′37" | 11.2 | 597 | *Vitex negundo* | 67.48 |
| 18 | E108°48′24" | N35°03′46" | 10.8 | 607 | *Ziziphus jujuba* | 57.63 |
| 19 | E108°48′36" | N35°03′40" | 11.6 | 592 | *Sophora davidii* | 97.66 |
| 20 | E110°27′40" | N38°50′36" | 7.7 | 440 | *Artemisia ordosica* | 95.64 |
| 21 | E110°07′34" | N38°44′23" | 7.1 | 428 | *Caragana korshinskii* | 76.74 |
| 22 | E110°14′37" | N38°47′16" | 9.0 | 437 | *Salix cheilophila* | 97.90 |
| 23 | E109°16′43" | N38°02′06" | 8.9 | 404 | *Caragana korshinskii* | 100.00 |
| 24 | E109°20′50" | N38°08′13" | 7.8 | 405 | *Salix cheilophila* | 89.73 |
| 25 | E108°50′12" | N37°40′24" | 8.2 | 399 | *Salix cheilophila* | 91.56 |
| 26 | E108°49′54" | N37°39′48" | 3.4 | 396 | *Artemisia ordosica* | 100.00 |
| 27 | E109°54′07″ | N38°29′29″ | 7.2 | 427 | *Artemisia ordosica* | 62.50 |
| 28 | E109°51′42″ | N38°30′08″ | 7.5 | 418 | *Caragana korshinskii* | 41.12 |

RB: The relative biomass of dominant species was calculated as the proportion of biomass of a dominant species to the total productivity of the community.
