# Supplementary material for: Phosphorus Limitation Governs N:P Stoichiometry in Semi‐Arid Shrublands: Evidence From Organ, Plant and Community Scales
Source: Ecol Evol. 2026 Apr 16;16(4):e73398. doi: 10.1002/ece3.73398 (PMC13087109; doi:10.1002/ece3.73398)
Supplement: Supplementary file 2 — Figure S1: Soil C:N:P stoichiometric characteristics and their correlations with temperature and precipitation. Figure S2:. The correlations among climate factors, community traits and soil nutrient stoichiometry. [file ECE3-16-e73398-s002.docx]

**Phosphorus Limitation Governs N:P Stoichiometry in Semi-Arid Shrublands: Evidence from Organ, Plant and Community Scales**

Yang Li^1,2^, Jiangchao Guo^3^, Yongfu Chai^3^, Mao Wang^3,4^, Pengcheng Wan^3^, Ming Yue^1,2,3*^

^1^*Xi’an Botanical Garden of Shaanxi Province,* *I**nstitute of Botany of Shaanxi Province, Xi’an 710061, China*

^2^*Shaanxi Engineering Research Centre for Conservation and Utilization of Botanical Resources, Xi’an 710061, China*

^3^*Key Laboratory of Resource Biology and Biotechnology in Western China (Ministry of Education),* *Northwest University, Xi’an 710069, China*

*^4^College of Grassland and Environment Sciences, Xinjiang Agricultural University, Urumchi, Xinjiang 830052, China*

*Corresponding author: [yueming@nwu.edu.cn](mailto:yueming@nwu.edu.cn)

**1. Supplementary Figures**

**
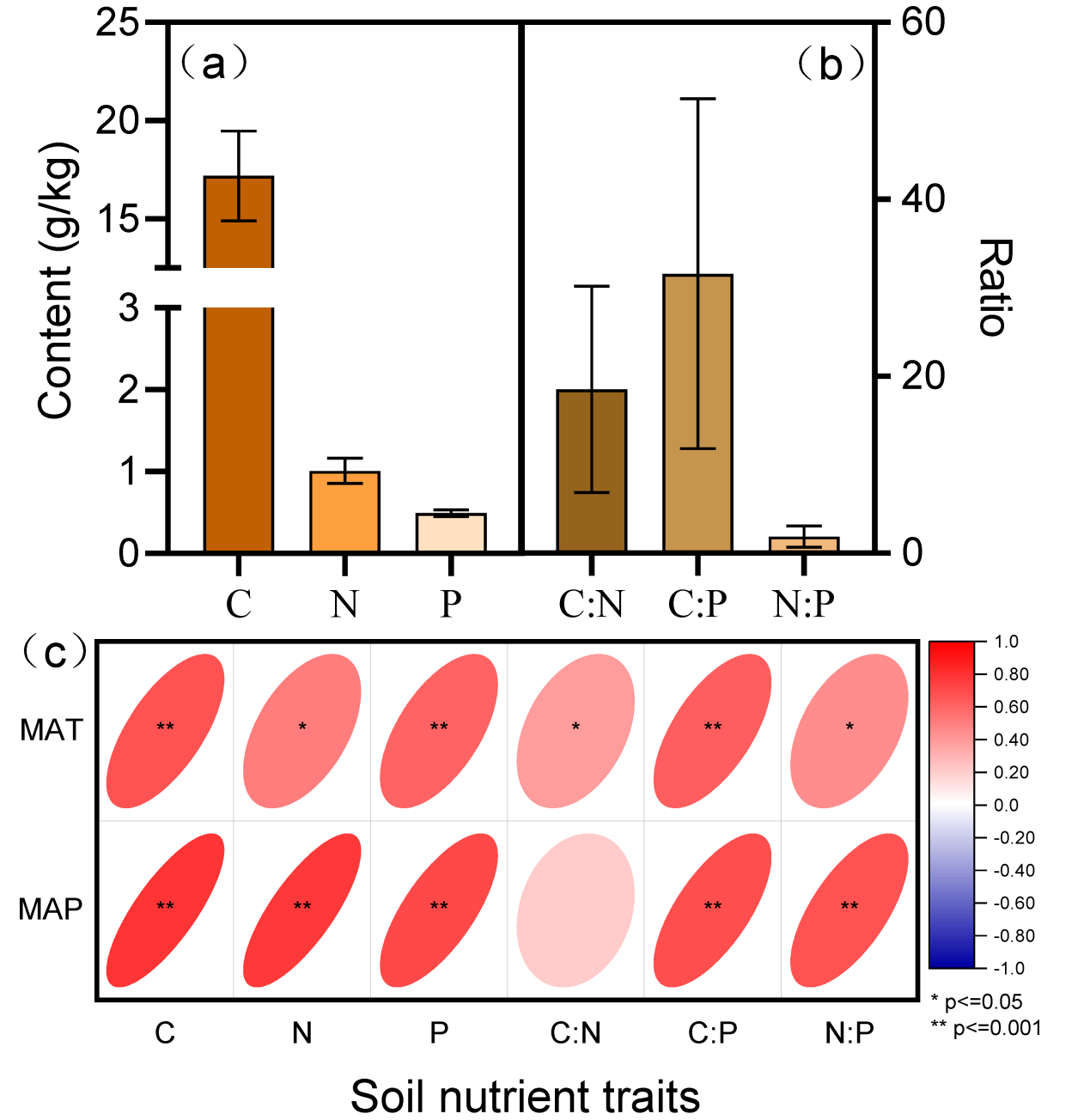
**

**Fig S1**. Soil C:N:P stoichiometric characteristics and their correlations with temperature and precipitation. (a) Soil C, N, and P contents; (b) Soil C:N:P ratios; (c) Correlations between soil C:N:P stoichiometric characteristics and mean annual temperature (MAT) and mean annual precipitation (MAP). ^*^: < 0.05, ^**^: <0.001.

**
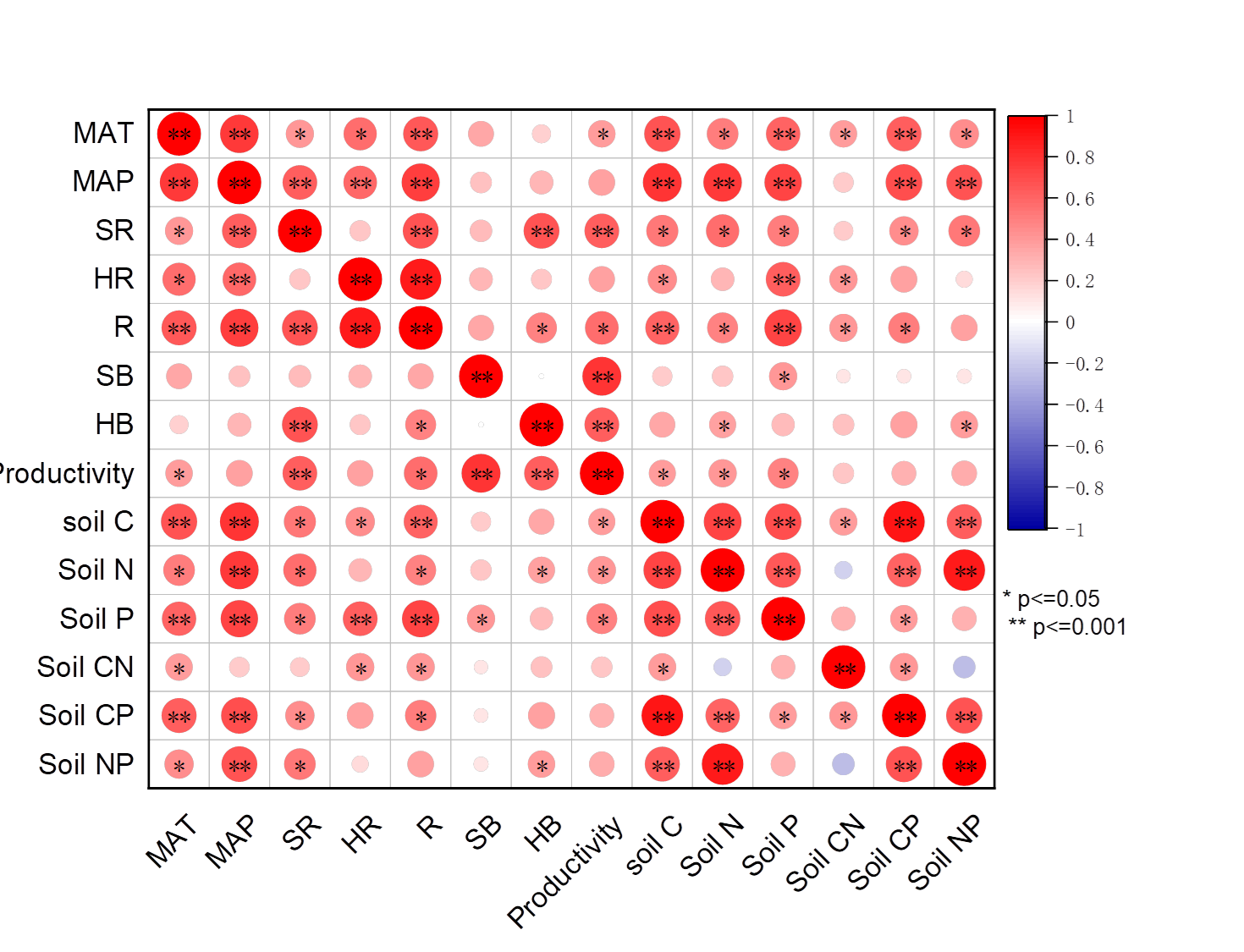
**

**Fig S2**. The correlations among climate factors, community traits and soil nutrient stoichiometry. MAT: mean annual temperature; MAP: mean annual precipitation; SR: shrub richness; HR: herb richness; R: richness; SB: shrub biomass; HB: herb biomass; Productivity: community productivity; Soil C: soil carbon content; Soil N: soil nitrogen content; Soil P: soil phosphorus content; Soil CN: soil carbon to nitrogen ratio, Soil CP: soil carbon to phosphorus ratio; Soil NP: soil nitrogen to phosphorus ratio. ^*^: < 0.05, ^**^: <0.001.
